# Supplementary material for: In Silico Research of New Therapeutics Rotenoids Derivatives against Leishmania amazonensis Infection
Source: Biology (Basel). 2022 Jan 14;11(1):133. doi: 10.3390/biology11010133 (PMC8772715; doi:10.3390/biology11010133)
Supplement: Supplementary file 1 [file biology-11-00133-s001.zip › biology-1532271-supplementary/Figure S1.pdf]

Figure S1. SwissTargetPrediction reports of the 20 main secondary metabolites.

SwissTargetPrediction report: 1,8-Cineol

Reference:
Gfeller D., Michielin O. & Zoete V.
Shaping the interaction landscape of bioactive molecules, *Bioinformatics* (2013) 29:3073-3079.

Query Molecule

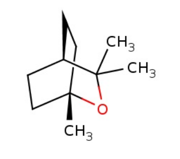

Frequency of Target Class

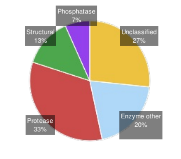

Target	Uniprot ID	Gene code	ChEMBL ID	Probability	# sim. cmpds (3D / 2D)	Target Class
Microtubule-associated protein tau	P10636	MAPT	CHEMBL1293224		1 / 4	Unclassified
Cytochrome P450 19A1	P11511	CYP19A1	CHEMBL1978		0 / 5	Enzyme
Desert hedgehog protein C-product *(by homology)*	O43323	DHH			0 / 10	Unclassified
Indian hedgehog protein N-product *(by homology)*	Q14623	IHH			0 / 10	Unclassified
Sonic hedgehog protein C-product *(by homology)*	Q15465	SHH	CHEMBL5602		0 / 10	Unclassified
Lanosterol 14-alpha demethylase *(by homology)*	Q16850	CYP51A1	CHEMBL3849		0 / 1	Enzyme
Cathepsin L1 light chain	P07711	CTSL1	CHEMBL3837		1 / 1	Cysteine Protease
Cathepsin B	P07858	CTSB	CHEMBL4072		1 / 1	Cysteine Protease
Cathepsin K	P43235	CTSK	CHEMBL268		1 / 1	Cysteine Protease
Cathepsin L2 *(by homology)*	O60911	CTSL2	CHEMBL3272		1 / 1	Cysteine Protease
Cathepsin S *(by homology)*	P25774	CTSS	CHEMBL2954		1 / 1	Cysteine Protease
Dynamin-1	Q05193	DNM1	CHEMBL4958		0 / 1	Structural
Dynamin-2 *(by homology)*	P50570	DNM2	CHEMBL5812		0 / 1	Enzyme
Dynamin-3 *(by homology)*	Q9UQ16	DNM3			0 / 1	Structural
M-phase inducer phosphatase 1	P30304	CDC25A	CHEMBL3775		0 / 10	Ser_Thr_Tyr Phosphatase

SwissTargetPrediction report: Artocarpin

Reference:
Gfeller D., Michielin O. & Zoete V.
Shaping the interaction landscape of bioactive molecules, *Bioinformatics* (2013) 29:3073-3079.

Query Molecule

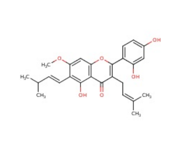

Frequency of Target Class

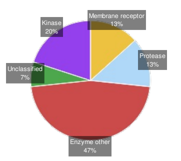

Target	Uniprot ID	Gene code	ChEMBL ID	Probability	# sim. cmpds (3D / 2D)	Target Class
Adenosine receptor A1 *(by homology)*	P30542	ADORA1	CHEMBL226		163 / 17	Membrane receptor
Adenosine receptor A3	P33765	ADORA3	CHEMBL256		106 / 15	Membrane receptor
Beta-secretase 1	P56817	BACE1	CHEMBL4822		24 / 13	Aspartic Protease
Beta-secretase 2 *(by homology)*	Q9Y5Z0	BACE2	CHEMBL2525		24 / 13	Aspartic Protease
Prostaglandin G/H synthase 1	P23219	PTGS1	CHEMBL221		24 / 7	Enzyme
Prostaglandin G/H synthase 2	P35354	PTGS2	CHEMBL230		24 / 7	Enzyme
ATP-binding cassette sub-family G member 2	Q9UNQ0	ABCG2	CHEMBL5393		11 / 19	Unclassified
cGMP-specific 3',5'-cyclic phosphodiesterase	O76074	PDE5A	CHEMBL1827		79 / 8	Enzyme
Dual 3',5'-cyclic-AMP and -GMP phosphodiesterase 11A *(by homology)*	Q9HCR9	PDE11A	CHEMBL2717		72 / 8	Enzyme
RAC-alpha serine/threonine-protein kinase	P31749	AKT1	CHEMBL4282		128 / 2	Ser_Thr Kinase
RAC-beta serine/threonine-protein kinase	P31751	AKT2	CHEMBL2431		128 / 2	Ser_Thr Kinase
RAC-gamma serine/threonine-protein kinase	Q9Y243	AKT3	CHEMBL4816		128 / 2	Ser_Thr Kinase
Arachidonate 5-lipoxygenase	P09917	ALOX5	CHEMBL215		43 / 45	Enzyme
Arachidonate 15-lipoxygenase *(by homology)*	P16050	ALOX15	CHEMBL2903		41 / 45	Enzyme
Arachidonate 12-lipoxygenase, 12S-type *(by homology)*	P18054	ALOX12	CHEMBL3687		41 / 45	Enzyme

SwissTargetPrediction report: Artoindesianin B

Reference:
Gfeller D., Michielin O. & Zoete V.
Shaping the interaction landscape of bioactive molecules, *Bioinformatics* (2013) 29:3073-3079.

Query Molecule

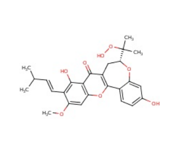

Frequency of Target Class

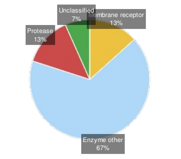

Target	Uniprot ID	Gene code	ChEMBL ID	Probability	# sim. cmpds (3D / 2D)	Target Class
Adenosine receptor A1	P30542	ADORA1	CHEMBL226		59 / 1	Membrane receptor
Adenosine receptor A3	P33765	ADORA3	CHEMBL256		31 / 3	Membrane receptor
Prostaglandin G/H synthase 1	P23219	PTGS1	CHEMBL221		3 / 1	Enzyme
Prostaglandin G/H synthase 2	P35354	PTGS2	CHEMBL230		3 / 1	Enzyme
Beta-secretase 1	P56817	BACE1	CHEMBL4822		9 / 7	Aspartic Protease
Beta-secretase 2 *(by homology)*	Q9Y5Z0	BACE2	CHEMBL2525		9 / 7	Aspartic Protease
cGMP-specific 3',5'-cyclic phosphodiesterase	O76074	PDE5A	CHEMBL1827		7 / 6	Enzyme
Dual 3',5'-cyclic-AMP and -GMP phosphodiesterase 11A *(by homology)*	Q9HCR9	PDE11A	CHEMBL2717		6 / 6	Enzyme
Arachidonate 5-lipoxygenase	P09917	ALOX5	CHEMBL215		6 / 33	Enzyme
Arachidonate 15-lipoxygenase *(by homology)*	P16050	ALOX15	CHEMBL2903		6 / 33	Enzyme
Arachidonate 15-lipoxygenase B *(by homology)*	O15296	ALOX15B	CHEMBL2457		6 / 33	Enzyme
Arachidonate 12-lipoxygenase, 12R-type *(by homology)*	O75342	ALOX12B			4 / 33	Enzyme
Arachidonate 12-lipoxygenase, 12S-type *(by homology)*	P18054	ALOX12	CHEMBL3687		6 / 33	Enzyme
Epidermis-type lipoxygenase 3 *(by homology)*	Q9BYJ1	ALOXE3			6 / 33	Enzyme
Microtubule-associated protein tau	P10636	MAPT	CHEMBL1293224		72 / 2	Unclassified

SwissTargetPrediction report: Artonin E

Reference:
Gfeller D., Michielin O. & Zoete V.
Shaping the interaction landscape of bioactive molecules, *Bioinformatics* (2013) 29:3073-3079.

Query Molecule

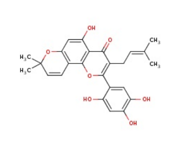

Frequency of Target Class

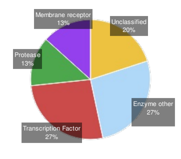

Target	Uniprot ID	Gene code	ChEMBL ID	Probability	# sim. cmpds (3D / 2D)	Target Class
ATP-binding cassette sub-family G member 2	Q9UNQ0	ABCG2	CHEMBL5393		13 / 2	Unclassified
Prostaglandin G/H synthase 1 *(by homology)*	P23219	PTGS1	CHEMBL221		13 / 1	Enzyme
Prostaglandin G/H synthase 2	P35354	PTGS2	CHEMBL230		13 / 1	Enzyme
Nuclear factor NF-kappa-B p50 subunit	P19838	NFKB1	CHEMBL3251		2 / 1	Transcription Factor
Transcription factor p65	Q04206	RELA	CHEMBL5533		1 / 2	Transcription Factor
Nuclear factor NF-kappa-B p100 subunit *(by homology)*	Q00653	NFKB2	CHEMBL3003		2 / 1	Transcription Factor
Proto-oncogene c-Rel *(by homology)*	Q04864	REL			1 / 2	Transcription Factor
Beta-secretase 1	P56817	BACE1	CHEMBL4822		15 / 2	Aspartic Protease
Beta-secretase 2 *(by homology)*	Q9Y5Z0	BACE2	CHEMBL2525		15 / 2	Aspartic Protease
Adenosine receptor A1	P30542	ADORA1	CHEMBL226		47 / 1	Membrane receptor
Adenosine receptor A3	P33765	ADORA3	CHEMBL256		17 / 3	Membrane receptor
Scavenger receptor class B member 1 *(by homology)*	Q8WTV0	SCARB1	CHEMBL1914272		1 / 1	Unclassified
Microtubule-associated protein tau	P10636	MAPT	CHEMBL1293224		203 / 5	Unclassified
Arachidonate 5-lipoxygenase	P09917	ALOX5	CHEMBL215		46 / 39	Enzyme
Arachidonate 15-lipoxygenase *(by homology)*	P16050	ALOX15	CHEMBL2903		47 / 39	Enzyme

SwissTargetPrediction report: Artonin V

Reference:
Gfeller D., Michielin O. & Zoete V.
Shaping the interaction landscape of bioactive molecules, *Bioinformatics* (2013) 29:3073-3079.

Query Molecule

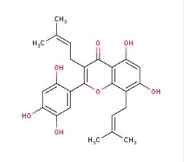

Frequency of Target Class

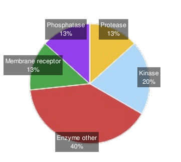

Target	Uniprot ID	Gene code	ChEMBL ID	Probability	# sim. cmpds (3D / 2D)	Target Class
Beta-secretase 1	P56817	BACE1	CHEMBL4822		8 / 12	Aspartic Protease
Beta-secretase 2 *(by homology)*	Q9Y5Z0	BACE2	CHEMBL2525		8 / 12	Aspartic Protease
RAC-alpha serine/threonine-protein kinase	P31749	AKT1	CHEMBL4282		110 / 2	Ser_Thr Kinase
RAC-beta serine/threonine-protein kinase *(by homology)*	P31751	AKT2	CHEMBL2431		110 / 2	Ser_Thr Kinase
RAC-gamma serine/threonine-protein kinase *(by homology)*	Q9Y243	AKT3	CHEMBL4816		110 / 2	Ser_Thr Kinase
cGMP-specific 3',5'-cyclic phosphodiesterase	O76074	PDE5A	CHEMBL1827		60 / 7	Enzyme
Dual 3',5'-cyclic-AMP and -GMP phosphodiesterase 11A *(by homology)*	Q9HCR9	PDE11A	CHEMBL2717		56 / 7	Enzyme
Adenosine receptor A3	P33765	ADORA3	CHEMBL256		13 / 15	Membrane receptor
Tyrosine-protein phosphatase non-receptor type 1	P18031	PTPN1	CHEMBL335		8 / 13	Tyr Phosphatase
Tyrosine-protein phosphatase non-receptor type 2 *(by homology)*	P17706	PTPN2	CHEMBL3807		8 / 13	Tyr Phosphatase
Adenosine receptor A1 *(by homology)*	P30542	ADORA1	CHEMBL226		29 / 18	Membrane receptor
Cytochrome P450 19A1	P11511	CYP19A1	CHEMBL1978		11 / 13	Enzyme
Arachidonate 5-lipoxygenase	P09917	ALOX5	CHEMBL215		55 / 46	Enzyme
Arachidonate 15-lipoxygenase *(by homology)*	P16050	ALOX15	CHEMBL2903		55 / 46	Enzyme
Arachidonate 12-lipoxygenase, 12S-type *(by homology)*	P18054	ALOX12	CHEMBL3687		55 / 46	Enzyme

SwissTargetPrediction report: Artonol B

Reference:
Gfeller D., Michielin O. & Zoete V.
Shaping the interaction landscape of bioactive molecules, *Bioinformatics* (2013) 29:3073-3079.

Query Molecule

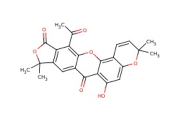

Frequency of Target Class

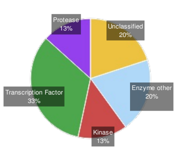

Target	Uniprot ID	Gene code	ChEMBL ID	Probability	# sim. cmpds (3D / 2D)	Target Class
ATP-binding cassette sub-family G member 2	Q9UNQ0	ABCG2	CHEMBL5393		11 / 1	Unclassified
Microtubule-associated protein tau	P10636	MAPT	CHEMBL1293224		604 / 3	Unclassified
Scavenger receptor class B member 1 *(by homology)*	Q8WTV0	SCARB1	CHEMBL1914272		2 / 1	Unclassified
Prostaglandin G/H synthase 1 *(by homology)*	P23219	PTGS1	CHEMBL221		28 / 2	Enzyme
Prostaglandin G/H synthase 2	P35354	PTGS2	CHEMBL230		28 / 2	Enzyme
Dual specificity tyrosine-phosphorylation-regulated kinase 1A *(by homology)*	Q13627	DYRK1A	CHEMBL2292		22 / 1	Ser_Thr_Tyr Kinase
Tyrosyl-DNA phosphodiesterase 1	Q9NUW8	TDP1	CHEMBL1075138		153 / 1	Enzyme
Nuclear factor NF-kappa-B p50 subunit	P19838	NFKB1	CHEMBL3251		1 / 1	Transcription Factor
Nuclear factor NF-kappa-B p100 subunit *(by homology)*	Q00653	NFKB2	CHEMBL3003		1 / 1	Transcription Factor
Beta-secretase 1	P56817	BACE1	CHEMBL4822		4 / 1	Aspartic Protease
Beta-secretase 2 *(by homology)*	Q9Y5Z0	BACE2	CHEMBL2525		4 / 1	Aspartic Protease
Peroxisome proliferator-activated receptor gamma	P37231	PPARG	CHEMBL235		2 / 3	Transcription Factor
Peroxisome proliferator-activated receptor alpha	Q07869	PPARA	CHEMBL239		2 / 3	Transcription Factor
Peroxisome proliferator-activated receptor delta *(by homology)*	Q03181	PPARD	CHEMBL3979		2 / 3	Transcription Factor
Protein kinase C gamma type *(by homology)*	P05129	PRKCG	CHEMBL2938		22 / 1	Ser_Thr Kinase

SwissTargetPrediction report: Bicyclogermacrene

Reference:
Gfeller D., Michielin O. & Zoete V.
Shaping the interaction landscape of bioactive molecules, *Bioinformatics* (2013) 29:3073-3079.

Query Molecule

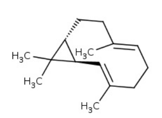

Frequency of Target Class

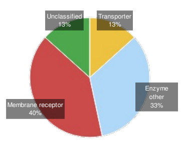

Target	Uniprot ID	Gene code	ChEMBL ID	Probability	# sim. cmpds (3D / 2D)	Target Class
Sodium-dependent noradrenaline transporter	P23975	SLC6A2	CHEMBL222		4 / 1	Transporter
Sodium-dependent dopamine transporter *(by homology)*	Q01959	SLC6A3	CHEMBL238		4 / 1	Transporter
Squalene monooxygenase *(by homology)*	Q14534	SQLE	CHEMBL3592		0 / 8	Enzyme
Tyrosyl-DNA phosphodiesterase 1	Q9NUW8	TDP1	CHEMBL1075138		1 / 4	Enzyme
Muscarinic acetylcholine receptor M2	P08172	CHRM2	CHEMBL211		1 / 1	Membrane receptor
Muscarinic acetylcholine receptor M4	P08173	CHRM4	CHEMBL1821		1 / 1	Membrane receptor
Muscarinic acetylcholine receptor M5	P08912	CHRM5	CHEMBL2035		1 / 1	Membrane receptor
Muscarinic acetylcholine receptor M1	P11229	CHRM1	CHEMBL216		1 / 1	Membrane receptor
Muscarinic acetylcholine receptor M3	P20309	CHRM3	CHEMBL245		1 / 1	Membrane receptor
Sigma non-opioid intracellular receptor 1	Q99720	SIGMAR1	CHEMBL287		12 / 1	Membrane receptor
Cytochrome P450 19A1	P11511	CYP19A1	CHEMBL1978		1 / 57	Enzyme
Acetylcholinesterase	P22303	ACHE	CHEMBL220		1 / 1	Enzyme
Cholinesterase *(by homology)*	P06276	BCHE	CHEMBL1914		1 / 1	Enzyme
Microtubule-associated protein tau	P10636	MAPT	CHEMBL1293224		1 / 2	Unclassified
Muscleblind-like protein 1	Q9NR56	MBNL1	CHEMBL1293317		3 / 1	Unclassified

SwissTargetPrediction report: Brunfelsamidine

Reference:
Gfeller D., Michielin O. & Zoete V.
Shaping the interaction landscape of bioactive molecules, *Bioinformatics* (2013) 29:3073-3079.

Query Molecule

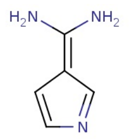

Frequency of Target Class

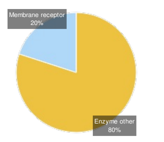

Target	Uniprot ID	Gene code	ChEMBL ID	Probability	# sim. cmpds (3D / 2D)	Target Class
Carbonic anhydrase 12	O43570	CA12	CHEMBL3242		2 / 0	Enzyme
Carbonic anhydrase 1	P00915	CA1	CHEMBL261		2 / 0	Enzyme
Carbonic anhydrase 2	P00918	CA2	CHEMBL205		2 / 0	Enzyme
Carbonic anhydrase 9	Q16790	CA9	CHEMBL3594		2 / 0	Enzyme
Carbonic anhydrase 14 *(by homology)*	Q9ULX7	CA14	CHEMBL3510		2 / 0	Enzyme
Carbonic anhydrase 3 *(by homology)*	P07451	CA3	CHEMBL2885		2 / 0	Enzyme
Carbonic anhydrase 5A, mitochondrial *(by homology)*	P35218	CA5A	CHEMBL4789		2 / 0	Enzyme
Carbonic anhydrase 7 *(by homology)*	P43166	CA7	CHEMBL2326		2 / 0	Enzyme
Carbonic anhydrase 13 *(by homology)*	Q8N1Q1	CA13	CHEMBL3912		2 / 0	Enzyme
Carbonic anhydrase 5B, mitochondrial *(by homology)*	Q9Y2D0	CA5B	CHEMBL3969		2 / 0	Enzyme
Adenosine receptor A2a	P29274	ADORA2A	CHEMBL251		6 / 0	Membrane receptor
Adenosine receptor A1 *(by homology)*	P30542	ADORA1	CHEMBL226		6 / 0	Membrane receptor
Adenosine receptor A2b *(by homology)*	P29275	ADORA2B	CHEMBL255		6 / 0	Membrane receptor
Nitric oxide synthase, endothelial	P29474	NOS3	CHEMBL4803		3 / 0	Enzyme
Nitric oxide synthase, brain *(by homology)*	P29475	NOS1	CHEMBL3568		3 / 0	Enzyme

SwissTargetPrediction report: 2,4-trans-4,5-trans-4,5-dihydroxypipecolic acid

Reference:
Gfeller D., Michielin O. & Zoete V.
Shaping the interaction landscape of bioactive molecules, *Bioinformatics* (2013) 29:3073-3079.

Query Molecule

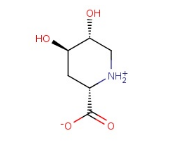

Frequency of Target Class

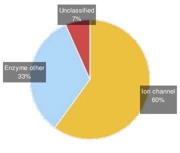

Target	Uniprot ID	Gene code	ChEMBL ID	Probability	# sim. cmpds (3D / 2D)	Target Class
Glutamate receptor ionotropic, kainate 1 *(by homology)*	P39086	GRIK1	CHEMBL1918		7 / 11	Ion channel
Glutamate receptor ionotropic, kainate 2 *(by homology)*	Q13002	GRIK2	CHEMBL3683		7 / 11	Ion channel
Glutamate receptor ionotropic, kainate 3 *(by homology)*	Q13003	GRIK3	CHEMBL3684		7 / 11	Ion channel
Glucoamylase	O43451	MGAM	CHEMBL2074		1 / 39	Enzyme
Lysosomal alpha-glucosidase	P10253	GAA	CHEMBL2608		1 / 18	Enzyme
Sucrase	P14410	SI	CHEMBL2748		1 / 39	Enzyme
Glucosylceramidase	P04062	GBA	CHEMBL2179		1 / 47	Enzyme
Glutamate receptor 1	P42261	GRIA1	CHEMBL2009		10 / 2	Ion channel
Glutamate receptor 2	P42262	GRIA2	CHEMBL4016		10 / 2	Ion channel
Glutamate receptor 3 *(by homology)*	P42263	GRIA3	CHEMBL3595		10 / 2	Ion channel
Glutamate receptor 4	P48058	GRIA4	CHEMBL3190		10 / 2	Ion channel
Microtubule-associated protein tau	P10636	MAPT	CHEMBL1293224		2 / 5	Unclassified
Glutamate receptor ionotropic, kainate 5	Q16478	GRIK5	CHEMBL2675		2 / 2	Ion channel
Glutamate receptor ionotropic, kainate 4 *(by homology)*	Q16099	GRIK4	CHEMBL2829		2 / 2	Ion channel
Tissue alpha-L-fucosidase	P04066	FUCA1	CHEMBL4176		0 / 37	Enzyme

SwissTargetPrediction report: Chaplasin

Reference:
Gfeller D., Michielin O. & Zoete V.
Shaping the interaction landscape of bioactive molecules, *Bioinformatics* (2013) 29:3073-3079.

Query Molecule

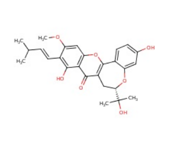

Frequency of Target Class

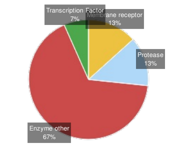

Target	Uniprot ID	Gene code	ChEMBL ID	Probability	# sim. cmpds (3D / 2D)	Target Class
Adenosine receptor A3	P33765	ADORA3	CHEMBL256		280 / 6	Membrane receptor
Adenosine receptor A1	P30542	ADORA1	CHEMBL226		367 / 3	Membrane receptor
Beta-secretase 1	P56817	BACE1	CHEMBL4822		59 / 11	Aspartic Protease
Beta-secretase 2 *(by homology)*	Q9Y5Z0	BACE2	CHEMBL2525		59 / 11	Aspartic Protease
Arachidonate 5-lipoxygenase	P09917	ALOX5	CHEMBL215		21 / 33	Enzyme
Arachidonate 15-lipoxygenase B *(by homology)*	O15296	ALOX15B	CHEMBL2457		21 / 33	Enzyme
Arachidonate 12-lipoxygenase, 12R-type *(by homology)*	O75342	ALOX12B			21 / 33	Enzyme
Arachidonate 15-lipoxygenase *(by homology)*	P16050	ALOX15	CHEMBL2903		21 / 33	Enzyme
Arachidonate 12-lipoxygenase, 12S-type *(by homology)*	P18054	ALOX12	CHEMBL3687		21 / 33	Enzyme
Epidermis-type lipoxygenase 3 *(by homology)*	Q9BYJ1	ALOXE3			21 / 33	Enzyme
Prostaglandin G/H synthase 1 *(by homology)*	P23219	PTGS1	CHEMBL221		11 / 2	Enzyme
Prostaglandin G/H synthase 2	P35354	PTGS2	CHEMBL230		11 / 2	Enzyme
cGMP-specific 3',5'-cyclic phosphodiesterase	O76074	PDE5A	CHEMBL1827		49 / 9	Enzyme
Dual 3',5'-cyclic-AMP and -GMP phosphodiesterase 11A *(by homology)*	Q9HCR9	PDE11A	CHEMBL2717		38 / 9	Enzyme
Hypoxia-inducible factor 1-alpha	Q16665	HIF1A	CHEMBL4261		6 / 15	Transcription Factor

SwissTargetPrediction report: Cycloaltilisin

Reference:
Gfeller D., Michielin O. & Zoete V.
Shaping the interaction landscape of bioactive molecules, *Bioinformatics* (2013) 29:3073-3079.

Query Molecule

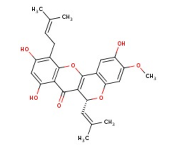

Frequency of Target Class

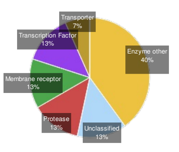

Target	Uniprot ID	Gene code	ChEMBL ID	Probability	# sim. cmpds (3D / 2D)	Target Class
Phospholipase A2	P04054	PLA2G1B	CHEMBL4426		7 / 1	Enzyme
Microtubule-associated protein tau	P10636	MAPT	CHEMBL1293224		513 / 6	Unclassified
Beta-secretase 1	P56817	BACE1	CHEMBL4822		17 / 4	Aspartic Protease
Beta-secretase 2 *(by homology)*	Q9Y5Z0	BACE2	CHEMBL2525		17 / 4	Aspartic Protease
ATP-binding cassette sub-family G member 2	Q9UNQ0	ABCG2	CHEMBL5393		23 / 8	Unclassified
Adenosine receptor A3	P33765	ADORA3	CHEMBL256		42 / 8	Membrane receptor
Prostaglandin G/H synthase 1	P23219	PTGS1	CHEMBL221		25 / 3	Enzyme
Prostaglandin G/H synthase 2	P35354	PTGS2	CHEMBL230		25 / 3	Enzyme
cGMP-specific 3',5'-cyclic phosphodiesterase	O76074	PDE5A	CHEMBL1827		127 / 7	Enzyme
Dual 3',5'-cyclic-AMP and -GMP phosphodiesterase 11A *(by homology)*	Q9HCR9	PDE11A	CHEMBL2717		116 / 7	Enzyme
Transcription factor p65	Q04206	RELA	CHEMBL5533		1 / 2	Transcription Factor
Proto-oncogene c-Rel *(by homology)*	Q04864	REL			1 / 2	Transcription Factor
Adenosine receptor A1	P30542	ADORA1	CHEMBL226		86 / 4	Membrane receptor
Multidrug resistance protein 1	P08183	ABCB1	CHEMBL4302		25 / 5	Transporter
Multidrug resistance protein 3 *(by homology)*	P21439	ABCB4	CHEMBL1743129		25 / 5	Enzyme

SwissTargetPrediction report: Cycloartenol

Reference:
Gfeller D., Michielin O. & Zoete V.
Shaping the interaction landscape of bioactive molecules, *Bioinformatics* (2013) 29:3073-3079.

Query Molecule

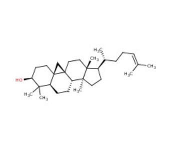

Frequency of Target Class

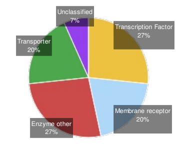

Target	Uniprot ID	Gene code	ChEMBL ID	Probability	# sim. cmpds (3D / 2D)	Target Class
Oxysterols receptor LXR-beta *(by homology)*	P55055	NR1H2	CHEMBL4093		11 / 23	Transcription Factor
Oxysterols receptor LXR-alpha	Q13133	NR1H3	CHEMBL2808		11 / 23	Transcription Factor
Low-density lipoprotein receptor	P01130	LDLR	CHEMBL3311		9 / 10	Membrane receptor
Very low-density lipoprotein receptor *(by homology)*	P98155	VLDLR			9 / 10	Membrane receptor
Low-density lipoprotein receptor-related protein 8 *(by homology)*	Q14114	LRP8			9 / 10	Membrane receptor
Cytochrome P450 19A1	P11511	CYP19A1	CHEMBL1978		3 / 78	Enzyme
Androgen receptor	P10275	AR	CHEMBL1871		7 / 75	Transcription Factor
Tyrosyl-DNA phosphodiesterase 1	Q9NUW8	TDP1	CHEMBL1075138		4 / 10	Enzyme
Sodium-dependent noradrenaline transporter	P23975	SLC6A2	CHEMBL222		17 / 6	Transporter
Sodium-dependent dopamine transporter *(by homology)*	Q01959	SLC6A3	CHEMBL238		17 / 6	Transporter
3-hydroxy-3-methylglutaryl-coenzyme A reductase	P04035	HMGCR	CHEMBL402		13 / 3	Enzyme
Lanosterol 14-alpha demethylase *(by homology)*	Q16850	CYP51A1	CHEMBL3849		2 / 2	Enzyme
Microtubule-associated protein tau	P10636	MAPT	CHEMBL1293224		2 / 8	Unclassified
Sodium-dependent serotonin transporter	P31645	SLC6A4	CHEMBL228		16 / 6	Transporter
Estrogen receptor	P03372	ESR1	CHEMBL206		2 / 50	Transcription Factor

SwissTargetPrediction report: Cycloartobiloxantone

Reference:
Gfeller D., Michielin O. & Zoete V.
Shaping the interaction landscape of bioactive molecules, *Bioinformatics* (2013) 29:3073-3079.

Query Molecule

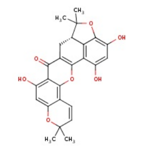

Frequency of Target Class

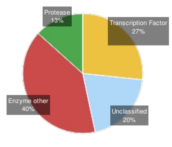

Target	Uniprot ID	Gene code	ChEMBL ID	Probability	# sim. cmpds (3D / 2D)	Target Class
Nuclear factor NF-kappa-B p50 subunit	P19838	NFKB1	CHEMBL3251		1 / 1	Transcription Factor
Transcription factor p65	Q04206	RELA	CHEMBL5533		1 / 2	Transcription Factor
Nuclear factor NF-kappa-B p100 subunit *(by homology)*	Q00653	NFKB2	CHEMBL3003		1 / 1	Transcription Factor
Proto-oncogene c-Rel *(by homology)*	Q04864	REL			1 / 2	Transcription Factor
ATP-binding cassette sub-family G member 2	Q9UNQ0	ABCG2	CHEMBL5393		9 / 1	Unclassified
Prostaglandin G/H synthase 1 *(by homology)*	P23219	PTGS1	CHEMBL221		4 / 1	Enzyme
Prostaglandin G/H synthase 2	P35354	PTGS2	CHEMBL230		4 / 1	Enzyme
Beta-secretase 1	P56817	BACE1	CHEMBL4822		8 / 1	Aspartic Protease
Beta-secretase 2 *(by homology)*	Q9Y5Z0	BACE2	CHEMBL2525		8 / 1	Aspartic Protease
Microtubule-associated protein tau	P10636	MAPT	CHEMBL1293224		123 / 3	Unclassified
Scavenger receptor class B member 1 *(by homology)*	Q8WTV0	SCARB1	CHEMBL1914272		1 / 1	Unclassified
Arachidonate 5-lipoxygenase	P09917	ALOX5	CHEMBL215		22 / 28	Enzyme
Arachidonate 15-lipoxygenase *(by homology)*	P16050	ALOX15	CHEMBL2903		22 / 28	Enzyme
Arachidonate 12-lipoxygenase, 12S-type *(by homology)*	P18054	ALOX12	CHEMBL3687		22 / 28	Enzyme
Arachidonate 15-lipoxygenase B *(by homology)*	O15296	ALOX15B	CHEMBL2457		22 / 28	Enzyme

SwissTargetPrediction report: Cycloartocarpin

Reference:
Gfeller D., Michielin O. & Zoete V.
Shaping the interaction landscape of bioactive molecules, *Bioinformatics* (2013) 29:3073-3079.

Query Molecule

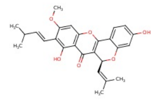

Frequency of Target Class

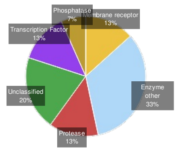

Target	Uniprot ID	Gene code	ChEMBL ID	Probability	# sim. cmpds (3D / 2D)	Target Class
Adenosine receptor A1 *(by homology)*	P30542	ADORA1	CHEMBL226		103 / 3	Membrane receptor
Adenosine receptor A3	P33765	ADORA3	CHEMBL256		62 / 6	Membrane receptor
Prostaglandin G/H synthase 1	P23219	PTGS1	CHEMBL221		20 / 2	Enzyme
Prostaglandin G/H synthase 2	P35354	PTGS2	CHEMBL230		20 / 2	Enzyme
Beta-secretase 1	P56817	BACE1	CHEMBL4822		16 / 4	Aspartic Protease
Beta-secretase 2 *(by homology)*	Q9Y5Z0	BACE2	CHEMBL2525		16 / 4	Aspartic Protease
ATP-binding cassette sub-family G member 2	Q9UNQ0	ABCG2	CHEMBL5393		12 / 5	Unclassified
Phospholipase A2	P04054	PLA2G1B	CHEMBL4426		4 / 1	Enzyme
Microtubule-associated protein tau	P10636	MAPT	CHEMBL1293224		571 / 4	Unclassified
cGMP-specific 3',5'-cyclic phosphodiesterase	O76074	PDE5A	CHEMBL1827		53 / 7	Enzyme
Dual 3',5'-cyclic-AMP and -GMP phosphodiesterase 11A *(by homology)*	Q9HCR9	PDE11A	CHEMBL2717		47 / 7	Enzyme
Transcription factor p65	Q04206	RELA	CHEMBL5533		2 / 2	Transcription Factor
Proto-oncogene c-Rel *(by homology)*	Q04864	REL			2 / 2	Transcription Factor
Scavenger receptor class B member 1 *(by homology)*	Q8WTV0	SCARB1	CHEMBL1914272		4 / 1	Unclassified
Tyrosine-protein phosphatase non-receptor type 2 *(by homology)*	P17706	PTPN2	CHEMBL3807		34 / 4	Tyr Phosphatase

SwissTargetPrediction report: D-Germacrene

Reference:
Gfeller D., Michielin O. & Zoete V.
Shaping the interaction landscape of bioactive molecules, *Bioinformatics* (2013) 29:3073-3079.

Query Molecule

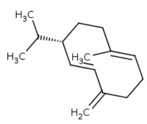

Frequency of Target Class

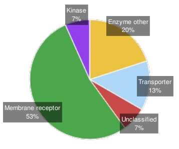

Target	Uniprot ID	Gene code	ChEMBL ID	Probability	# sim. cmpds (3D / 2D)	Target Class
Cytochrome P450 19A1	P11511	CYP19A1	CHEMBL1978		1 / 41	Enzyme
Fatty-acid amide hydrolase 1	O00519	FAAH	CHEMBL2243		1 / 9	Enzyme
Sodium-dependent noradrenaline transporter	P23975	SLC6A2	CHEMBL222		11 / 1	Transporter
Sodium-dependent dopamine transporter *(by homology)*	Q01959	SLC6A3	CHEMBL238		11 / 1	Transporter
Microtubule-associated protein tau	P10636	MAPT	CHEMBL1293224		7 / 1	Unclassified
Alpha-2A adrenergic receptor	P08913	ADRA2A	CHEMBL1867		43 / 1	Membrane receptor
Alpha-2B adrenergic receptor	P18089	ADRA2B	CHEMBL1942		43 / 1	Membrane receptor
Alpha-2C adrenergic receptor	P18825	ADRA2C	CHEMBL1916		43 / 1	Membrane receptor
Tyrosyl-DNA phosphodiesterase 1	Q9NUW8	TDP1	CHEMBL1075138		4 / 1	Enzyme
Muscarinic acetylcholine receptor M2	P08172	CHRM2	CHEMBL211		2 / 1	Membrane receptor
Muscarinic acetylcholine receptor M4	P08173	CHRM4	CHEMBL1821		2 / 1	Membrane receptor
Muscarinic acetylcholine receptor M5	P08912	CHRM5	CHEMBL2035		1 / 1	Membrane receptor
Muscarinic acetylcholine receptor M1	P11229	CHRM1	CHEMBL216		2 / 1	Membrane receptor
Muscarinic acetylcholine receptor M3	P20309	CHRM3	CHEMBL245		1 / 1	Membrane receptor
Mitogen-activated protein kinase 14	Q16539	MAPK14	CHEMBL260		2 / 1	Ser_Thr Kinase

SwissTargetPrediction report: Deguelin

Reference:
Gfeller D., Michielin O. & Zoete V.
Shaping the interaction landscape of bioactive molecules, *Bioinformatics* (2013) 29:3073-3079.

Query Molecule

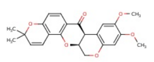

Frequency of Target Class

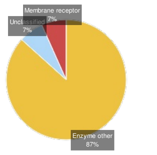

Target	Uniprot ID	Gene code	ChEMBL ID	Probability	# sim. cmpds (3D / 2D)	Target Class
Tyrosyl-DNA phosphodiesterase 1	Q9NUW8	TDP1	CHEMBL1075138		220 / 20	Enzyme
Ornithine decarboxylase	P11926	ODC1	CHEMBL1869		1 / 10	Enzyme
Antizyme inhibitor 1 *(by homology)*	O14977	AZIN1			1 / 10	Enzyme
Arginine decarboxylase *(by homology)*	Q96A70	ADC			1 / 10	Enzyme
Microtubule-associated protein tau	P10636	MAPT	CHEMBL1293224		1904 / 25	Unclassified
NADH-ubiquinone oxidoreductase chain 4	P03905	MT-ND4	CHEMBL4499		5 / 5	Enzyme
Cytochrome P450 2C19	P33261	CYP2C19	CHEMBL3622		2 / 1	Enzyme
5-hydroxytryptamine receptor 6	P50406	HTR6	CHEMBL3371		9 / 1	Membrane receptor
Cytochrome P450 2E1 *(by homology)*	P05181	CYP2E1	CHEMBL5281		2 / 1	Enzyme
Cytochrome P450 2C8 *(by homology)*	P10632	CYP2C8	CHEMBL3721		2 / 1	Enzyme
Cytochrome P450 2A6 *(by homology)*	P11509	CYP2A6	CHEMBL5282		2 / 1	Enzyme
Cytochrome P450 2C9 *(by homology)*	P11712	CYP2C9	CHEMBL3397		2 / 1	Enzyme
Cytochrome P450 2B6 *(by homology)*	P20813	CYP2B6	CHEMBL4729		2 / 1	Enzyme
Cytochrome P450 2A7 *(by homology)*	P20853	CYP2A7			2 / 1	Enzyme
Cytochrome P450 2F1 *(by homology)*	P24903	CYP2F1			2 / 1	Enzyme

Target	Uniprot ID	Gene Code	ChEMBL ID	By Homology	Probability	Number of sim. cmpds (3D)	Number of sim. cmpds (D2)	Target Class
Tyrosyl-DNA phosphodiesterase 1	Q9NUW8	TDP1	CHEMBL1075138	No	0.92	220	20	Enzyme
Ornithine decarboxylase	P11926	ODC1	CHEMBL1869	No	0.86	1	10	Enzyme
Antizyme inhibitor 1	O14977	AZIN1		Yes	0.86	1	10	Enzyme
Arginine decarboxylase	Q96A70	ADC		Yes	0.86	1	10	Enzyme
Microtubule-associated protein tau	P10636	MAPT	CHEMBL1293224	No	0.60	1904	25	Unclassified
NADH-ubiquinone oxidoreductase chain 4	P03905	MT-ND4	CHEMBL4499	No	0.49	5	5	Enzyme
Cytochrome P450 2C19	P33261	CYP2C19	CHEMBL3622	No	0.49	2	1	Enzyme
5-hydroxytryptamine receptor 6	P50406	HTR6	CHEMBL3371	No	0.49	9	1	Membrane receptor
Cytochrome P450 2E1	P05181	CYP2E1	CHEMBL5281	Yes	0.49	2	1	Enzyme
Cytochrome P450 2C8	P10632	CYP2C8	CHEMBL3721	Yes	0.49	2	1	Enzyme
Cytochrome P450 2A6	P11509	CYP2A6	CHEMBL5282	Yes	0.49	2	1	Enzyme
Cytochrome P450 2C9	P11712	CYP2C9	CHEMBL3397	Yes	0.49	2	1	Enzyme
Cytochrome P450 2B6	P20813	CYP2B6	CHEMBL4729	Yes	0.49	2	1	Enzyme
Cytochrome P450 2A7	P20853	CYP2A7		Yes	0.49	2	1	Enzyme
Cytochrome P450 2F1	P24903	CYP2F1		Yes	0.49	2	1	Enzyme

SwissTargetPrediction report: Escopoletin

Reference:
Gfeller D., Michielin O. & Zoete V.
Shaping the interaction landscape of bioactive molecules, *Bioinformatics* (2013) 29:3073-3079.

Query Molecule

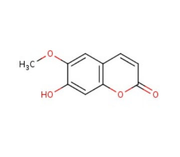

Frequency of Target Class

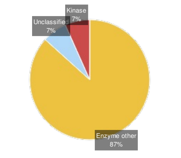

Target	Uniprot ID	Gene code	ChEMBL ID	Probability	# sim. cmpds (3D / 2D)	Target Class
FAD-linked sulfhydryl oxidase ALR	P55789	GFER	CHEMBL1741189		6 / 6	Enzyme
Carbonic anhydrase 9	Q16790	CA9	CHEMBL3594		5 / 47	Enzyme
Carbonic anhydrase 1	P00915	CA1	CHEMBL261		25 / 28	Enzyme
Carbonic anhydrase 2 *(by homology)*	P00918	CA2	CHEMBL205		25 / 28	Enzyme
Carbonic anhydrase 3 *(by homology)*	P07451	CA3	CHEMBL2885		25 / 28	Enzyme
Carbonic anhydrase 5A, mitochondrial	P35218	CA5A	CHEMBL4789		25 / 28	Enzyme
Carbonic anhydrase 7	P43166	CA7	CHEMBL2326		25 / 28	Enzyme
Carbonic anhydrase 13 *(by homology)*	Q8N1Q1	CA13	CHEMBL3912		25 / 28	Enzyme
Carbonic anhydrase 5B, mitochondrial	Q9Y2D0	CA5B	CHEMBL3969		25 / 28	Enzyme
Microtubule-associated protein tau	P10636	MAPT	CHEMBL1293224		10 / 42	Unclassified
Carbonic anhydrase 12	O43570	CA12	CHEMBL3242		3 / 30	Enzyme
Carbonic anhydrase 14	Q9ULX7	CA14	CHEMBL3510		3 / 30	Enzyme
Carbonic anhydrase 4	P22748	CA4	CHEMBL3729		11 / 7	Enzyme
Dual specificity tyrosine-phosphorylation-regulated kinase 1A *(by homology)*	Q13627	DYRK1A	CHEMBL2292		5 / 24	Ser_Thr_Tyr Kinase
Carbonic anhydrase 6	P23280	CA6	CHEMBL3025		2 / 8	Enzyme

SwissTargetPrediction report: Fenchone

Reference:
Gfeller D., Michielin O. & Zoete V.
Shaping the interaction landscape of bioactive molecules, *Bioinformatics* (2013) 29:3073-3079.

Query Molecule

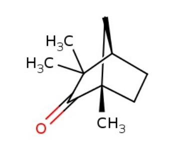

Frequency of Target Class

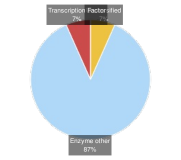

Target	Uniprot ID	Gene code	ChEMBL ID	Probability	# sim. cmpds (3D / 2D)	Target Class
Microtubule-associated protein tau	P10636	MAPT	CHEMBL1293224		5 / 3	Unclassified
Cytochrome P450 19A1	P11511	CYP19A1	CHEMBL1978		14 / 30	Enzyme
Carbonic anhydrase 1	P00915	CA1	CHEMBL261		2 / 2	Enzyme
Carbonic anhydrase 2	P00918	CA2	CHEMBL205		2 / 2	Enzyme
Carbonic anhydrase 4	P22748	CA4	CHEMBL3729		1 / 1	Enzyme
Carbonic anhydrase 3 *(by homology)*	P07451	CA3	CHEMBL2885		2 / 2	Enzyme
Carbonic anhydrase 5A, mitochondrial *(by homology)*	P35218	CA5A	CHEMBL4789		2 / 2	Enzyme
Carbonic anhydrase 7 *(by homology)*	P43166	CA7	CHEMBL2326		2 / 2	Enzyme
Carbonic anhydrase 13 *(by homology)*	Q8N1Q1	CA13	CHEMBL3912		2 / 2	Enzyme
Carbonic anhydrase 5B, mitochondrial *(by homology)*	Q9Y2D0	CA5B	CHEMBL3969		2 / 2	Enzyme
Androgen receptor	P10275	AR	CHEMBL1871		5 / 9	Transcription Factor
Tyrosyl-DNA phosphodiesterase 1	Q9NUW8	TDP1	CHEMBL1075138		2 / 4	Enzyme
Testosterone 17-beta-dehydrogenase 3	P37058	HSD17B3	CHEMBL4234		2 / 11	Enzyme
Estradiol 17-beta-dehydrogenase 12 *(by homology)*	Q53GQ0	HSD17B12	CHEMBL5998		2 / 11	Enzyme
Corticosteroid 11-beta-dehydrogenase isozyme 1	P28845	HSD11B1	CHEMBL4235		13 / 3	Enzyme

SwissTargetPrediction report: Morusin

Reference:
Gfeller D., Michielin O. & Zoete V.
Shaping the interaction landscape of bioactive molecules, *Bioinformatics* (2013) 29:3073-3079.

Query Molecule

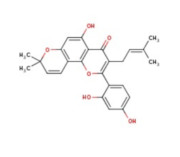

Frequency of Target Class

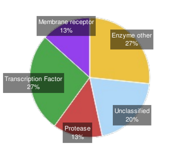

Target	Uniprot ID	Gene code	ChEMBL ID	Probability	# sim. cmpds (3D / 2D)	Target Class
Prostaglandin G/H synthase 1 *(by homology)*	P23219	PTGS1	CHEMBL221		17 / 1	Enzyme
Prostaglandin G/H synthase 2	P35354	PTGS2	CHEMBL230		17 / 1	Enzyme
ATP-binding cassette sub-family G member 2	Q9UNQ0	ABCG2	CHEMBL5393		18 / 3	Unclassified
Beta-secretase 1	P56817	BACE1	CHEMBL4822		21 / 8	Aspartic Protease
Beta-secretase 2 *(by homology)*	Q9Y5Z0	BACE2	CHEMBL2525		21 / 8	Aspartic Protease
Nuclear factor NF-kappa-B p50 subunit	P19838	NFKB1	CHEMBL3251		2 / 1	Transcription Factor
Transcription factor p65	Q04206	RELA	CHEMBL5533		3 / 2	Transcription Factor
Nuclear factor NF-kappa-B p100 subunit *(by homology)*	Q00653	NFKB2	CHEMBL3003		2 / 1	Transcription Factor
Proto-oncogene c-Rel *(by homology)*	Q04864	REL			3 / 2	Transcription Factor
Adenosine receptor A1 *(by homology)*	P30542	ADORA1	CHEMBL226		64 / 1	Membrane receptor
Adenosine receptor A3	P33765	ADORA3	CHEMBL256		33 / 3	Membrane receptor
Microtubule-associated protein tau	P10636	MAPT	CHEMBL1293224		326 / 3	Unclassified
Scavenger receptor class B member 1	Q8WTV0	SCARB1	CHEMBL1914272		2 / 1	Unclassified
cGMP-specific 3',5'-cyclic phosphodiesterase	O76074	PDE5A	CHEMBL1827		91 / 7	Enzyme
Dual 3',5'-cyclic-AMP and -GMP phosphodiesterase 11A *(by homology)*	Q9HCR9	PDE11A	CHEMBL2717		80 / 7	Enzyme

SwissTargetPrediction report: Rotenone

Reference:
Gfeller D., Michielin O. & Zoete V.
Shaping the interaction landscape of bioactive molecules, *Bioinformatics* (2013) 29:3073-3079.

Query Molecule

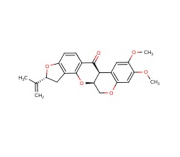

Frequency of Target Class

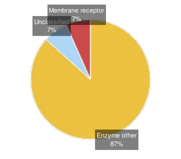

Target	Uniprot ID	Gene code	ChEMBL ID	Probability	# sim. cmpds (3D / 2D)	Target Class
NADH-ubiquinone oxidoreductase chain 4	P03905	MT-ND4	CHEMBL4499		5 / 5	Enzyme
Microtubule-associated protein tau	P10636	MAPT	CHEMBL1293224		662 / 27	Unclassified
Cytochrome P450 2C19	P33261	CYP2C19	CHEMBL3622		1 / 1	Enzyme
5-hydroxytryptamine receptor 6	P50406	HTR6	CHEMBL3371		2 / 1	Membrane receptor
Tyrosyl-DNA phosphodiesterase 1	Q9NUW8	TDP1	CHEMBL1075138		68 / 20	Enzyme
Cytochrome P450 2E1 *(by homology)*	P05181	CYP2E1	CHEMBL5281		1 / 1	Enzyme
Cytochrome P450 2C8 *(by homology)*	P10632	CYP2C8	CHEMBL3721		1 / 1	Enzyme
Cytochrome P450 2A6 *(by homology)*	P11509	CYP2A6	CHEMBL5282		1 / 1	Enzyme
Cytochrome P450 2C9 *(by homology)*	P11712	CYP2C9	CHEMBL3397		1 / 1	Enzyme
Cytochrome P450 2B6 *(by homology)*	P20813	CYP2B6	CHEMBL4729		1 / 1	Enzyme
Cytochrome P450 2A7 *(by homology)*	P20853	CYP2A7			1 / 1	Enzyme
Cytochrome P450 2F1 *(by homology)*	P24903	CYP2F1			1 / 1	Enzyme
Cytochrome P450 2C18 *(by homology)*	P33260	CYP2C18	CHEMBL2408		1 / 1	Enzyme
Cytochrome P450 2A13 *(by homology)*	Q16696	CYP2A13			1 / 1	Enzyme
Cytochrome P450 19A1	P11511	CYP19A1	CHEMBL1978		22 / 34	Enzyme
