# Supplementary material for: In Silico Research of New Therapeutics Rotenoids Derivatives against Leishmania amazonensis Infection
Source: Biology (Basel). 2022 Jan 14;11(1):133. doi: 10.3390/biology11010133 (PMC8772715; doi:10.3390/biology11010133)
Supplement: Supplementary file 1 [file biology-11-00133-s001.zip › biology-1532271-supplementary/Table S2.pdf]

TOP	LIGAND	SMILES	5XTD AFFINITY (KCAL/MOL)	ODC AFFINITY (KCAL/MOL)	RATIO
1	12.11_14	O1C2C(c3c(OC2)cc(OC)c(OC)c3)(C(=O)c2c1c1c(OC(C=C1)(C)C)cc2)CCCC(=O)N	-7,203	-10,646	1,47799528
2	12.6_7	O1C2C(c3c(OC2)cc(OC)c(OC)c3)(C(=O)c2c1c1c(OC(C=C1)(C)C)cc2)C(=O)NCl	-7,129	-10,479	1,46991163
3	12.6_17	O1C2C(c3c(OC2)cc(OC)c(OC)c3)(C(=O)c2c1c1c(OC(C=C1)(C)C)cc2)C(=O)NC(F)(F)F	-7,233	-10,551	1,45873082
4	12.6_1	O1C2C(c3c(OC2)cc(OC)c(OC)c3)(C(=O)c2c1c1c(OC(C=C1)(C)C)cc2)C(=O)NC	-7,101	-10,335	1,45542881
5	12.6_14	O1C2C(c3c(OC2)cc(OC)c(OC)c3)(C(=O)c2c1c1c(OC(C=C1)(C)C)cc2)C(=O)NC(=O)N	-7,059	-10,232	1,4494971
6	6.3_10	O1C2C(c3c(OC2)cc(OC)c(OOCO)c3)C(=O)c2c1c1c(OC(C=C1)(C)C)cc2	-6,816	-9,873	1,44850352
7	12.14_19	O1C2C(c3c(OC2)cc(OC)c(OC)c3)(C(=O)c2c1c1c(OC(C=C1)(C)C)cc2)NCCC(=O)O	-6,961	-10,079	1,44792415
8	12.19_2	O1C2C(c3c(OC2)cc(OC)c(OC)c3)(C(=O)c2c1c1c(OC(C=C1)(C)C)cc2)c1cccc(c1)O	-7,6	-11	1,44736842
9	12.16_6	O1C2C(c3c(OC2)cc(OC)c(OC)c3)(C(=O)c2c1c1c(OC(C=C1)(C)C)cc2)c1c(cccc1)OC	-7,778	-11,256	1,44715865
10	12.9_13	O1C2C(c3c(OC2)cc(OC)c(OC)c3)(C(=O)c2c1c1c(OC(C=C1)(C)C)cc2)C(=O)OCc1ccccc1	-7,733	-11,182	1,4460106
11	9.20_1	O1C2C(c3c(OC2c2ccc(CC)cc2)cc(OC)c(OC)c3)C(=O)c2c1c1c(OC(C=C1)(C)C)cc2	-7,823	-11,3	1,44445865
12	12.11_19	O1C2C(c3c(OC2)cc(OC)c(OC)c3)(C(=O)c2c1c1c(OC(C=C1)(C)C)cc2)CCCCC(=O)O	-7,243	-10,459	1,44401491
13	12.19_19	O1C2C(c3c(OC2)cc(OC)c(OC)c3)(C(=O)c2c1c1c(OC(C=C1)(C)C)cc2)c1cccc(c1)CC(=O)O	-7,561	-10,907	1,44253406
14	12.6_9	O1C2C(c3c(OC2)cc(OC)c(OC)c3)(C(=O)c2c1c1c(OC(C=C1)(C)C)cc2)C(=O)NCC	-7,176	-10,342	1,44119287
15	12.19_16	O1C2C(c3c(OC2)cc(OC)c(OC)c3)(C(=O)c2c1c1c(OC(C=C1)(C)C)cc2)c1cccc(c1)C#N	-7,568	-10,872	1,43657505
16	12.11_8	O1C2C(c3c(OC2)cc(OC)c(OC)c3)(C(=O)c2c1c1c(OC(C=C1)(C)C)cc2)CCCF	-7,311	-10,499	1,43605526
17	12.5_2	O1C2C(c3c(OC2)cc(OC)c(OC)c3)(C(=O)c2c1c1c(OC(C=C1)(C)C)cc2)c1ccc(cc1)O	-7,655	-10,991	1,4357936
18	6.18_19	O1C2C(c3c(OC2)cc(OC)c(OC(C(=O)O)CC(=O)O)c3)C(=O)c2c1c1c(OC(C=C1)(C)C)cc2	-6,751	-9,693	1,43578729
19	12.11_7	O1C2C(c3c(OC2)cc(OC)c(OC)c3)(C(=O)c2c1c1c(OC(C=C1)(C)C)cc2)CCCCl	-7,37	-10,569	1,43405699
20	9.20_16	O1C2C(c3c(OC2c2ccc(CC#N)cc2)cc(OC)c(OC)c3)C(=O)c2c1c1c(OC(C=C1)(C)C)cc2	-7,774	-11,14	1,43298173
21	9.5_1	O1C2C(c3c(OC2c2ccc(cc2)C)cc(OC)c(OC)c3)C(=O)c2c1c1c(OC(C=C1)(C)C)cc2	-7,757	-11,111	1,43238365
22	12.19_8	O1C2C(c3c(OC2)cc(OC)c(OC)c3)(C(=O)c2c1c1c(OC(C=C1)(C)C)cc2)c1cccc(c1)F	-7,746	-11,085	1,43106119
23	12.18_2	O1C2C(c3c(OC2)cc(OC)c(OC)c3)(C(=O)c2c1c1c(OC(C=C1)(C)C)cc2)C(C(=O)O)O	-6,845	-9,793	1,43067933
24	12.19_7	O1C2C(c3c(OC2)cc(OC)c(OC)c3)(C(=O)c2c1c1c(OC(C=C1)(C)C)cc2)c1cccc(c1)Cl	-7,825	-11,186	1,42952077
25	6.12_10	O1C2C(c3c(OC2)cc(OC)c(OOCCO)c3)C(=O)c2c1c1c(OC(C=C1)(C)C)cc2	-7,083	-10,123	1,42919667
