# Supplementary figures and images for: In Silico Research of New Therapeutics Rotenoids Derivatives against Leishmania amazonensis Infection
Source: Biology (Basel). 2022 Jan 14;11(1):133. doi: 10.3390/biology11010133 (PMC8772715; doi:10.3390/biology11010133)

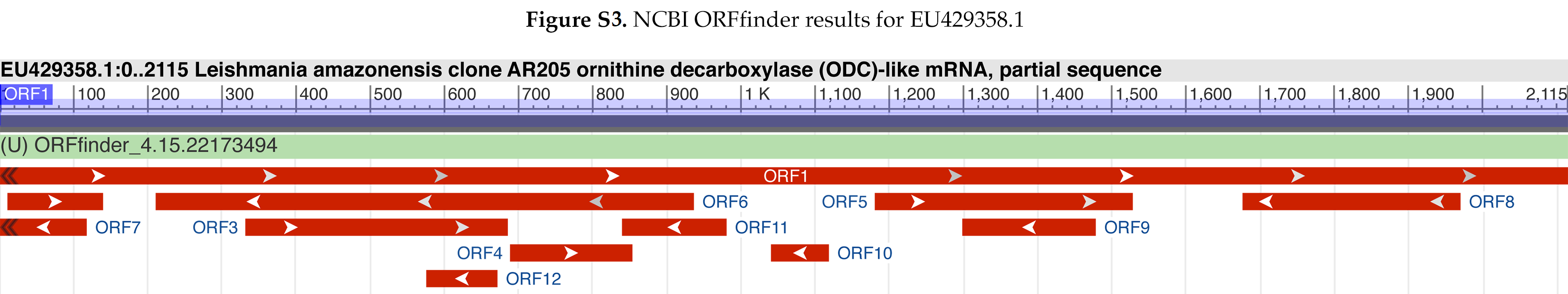

Supplement: Supplementary file 1 [file biology-11-00133-s001.zip › biology-1532271-supplementary/Figure S3.tif]

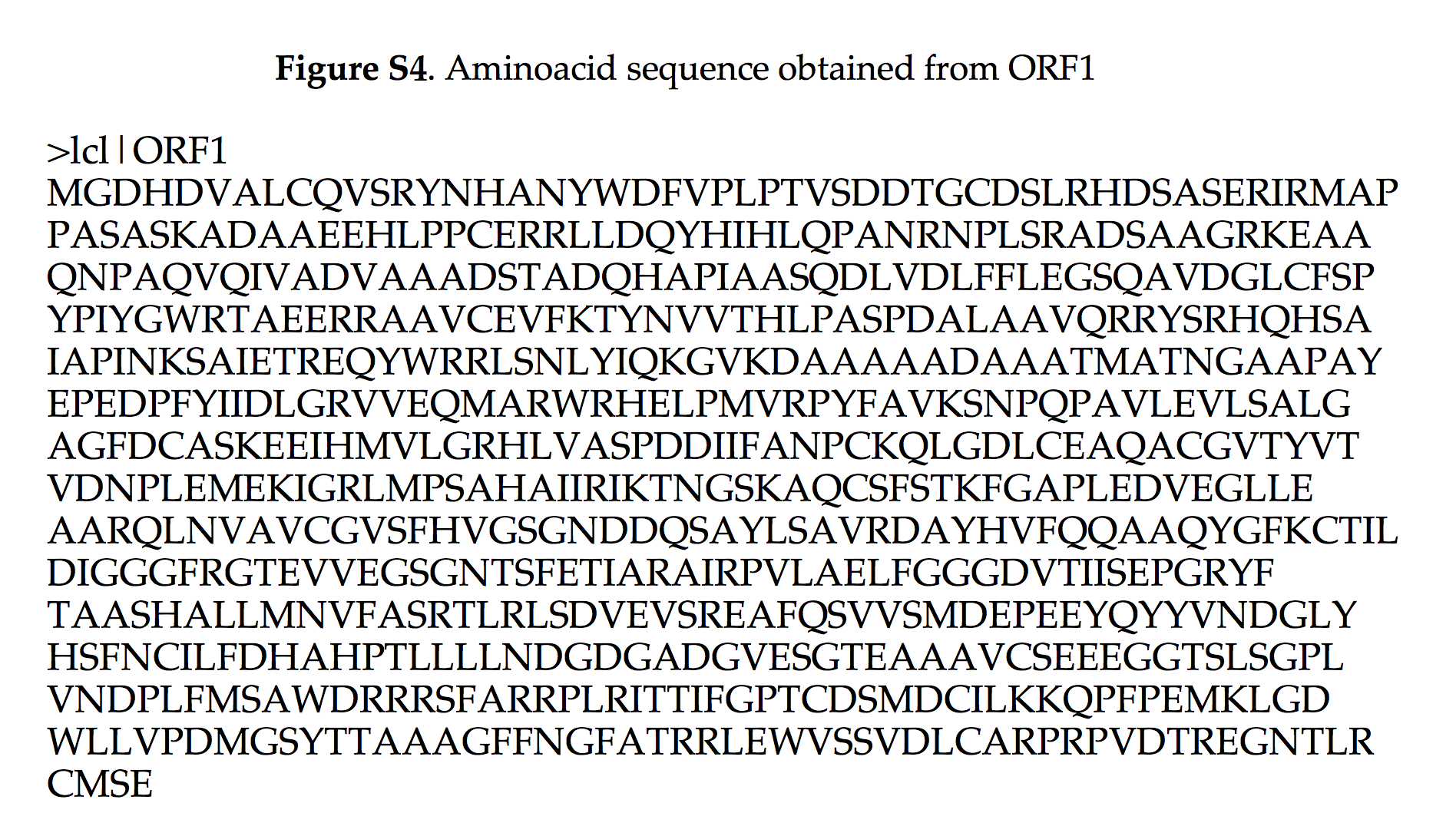

Supplement: Supplementary file 1 [file biology-11-00133-s001.zip › biology-1532271-supplementary/Figure S4.tiff]

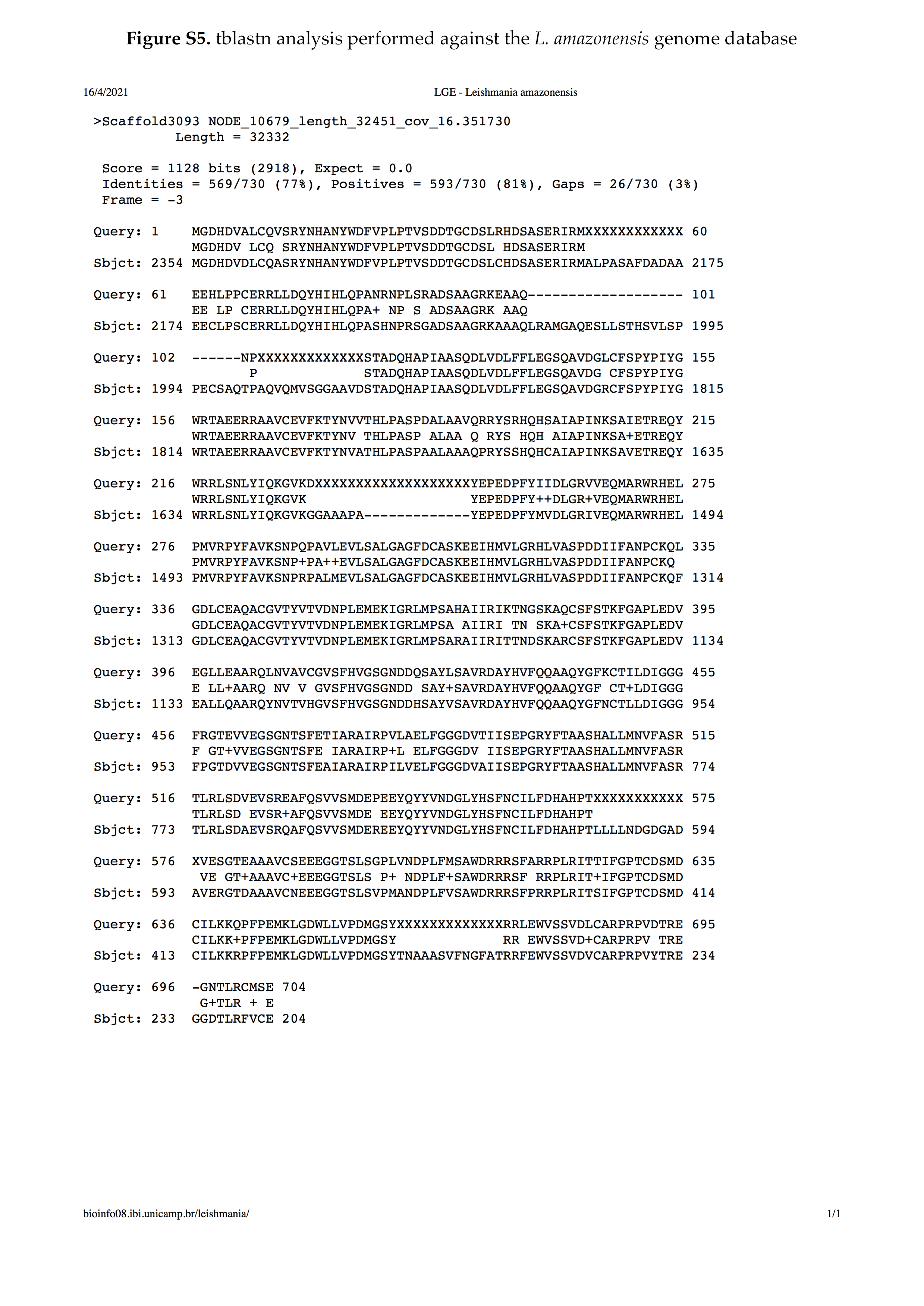

Supplement: Supplementary file 1 [file biology-11-00133-s001.zip › biology-1532271-supplementary/Figure S5.tiff]

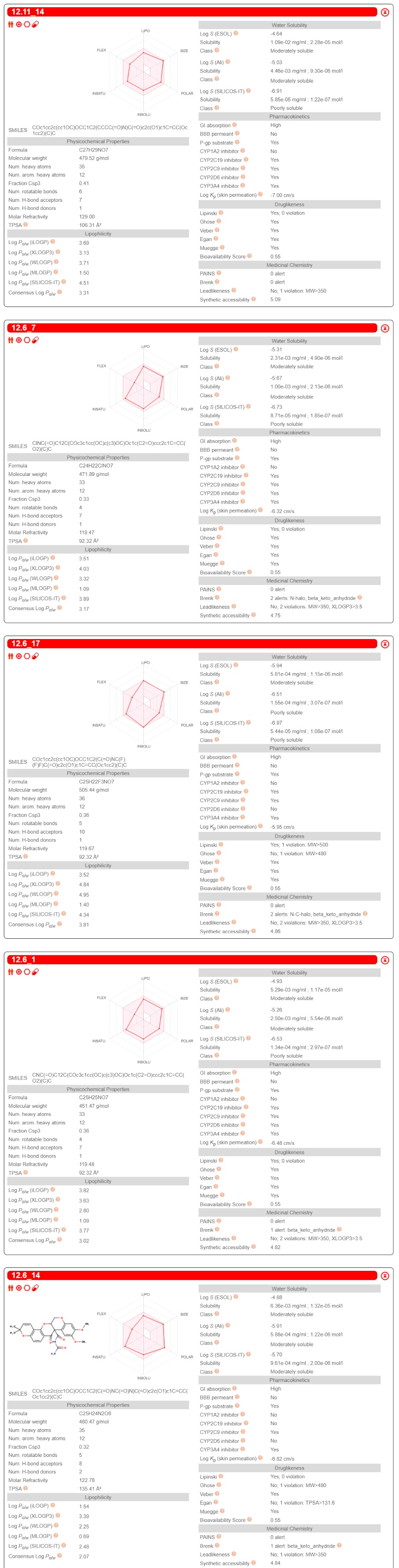

Supplement: Supplementary file 1 [file biology-11-00133-s001.zip › biology-1532271-supplementary/Figure S6.png]

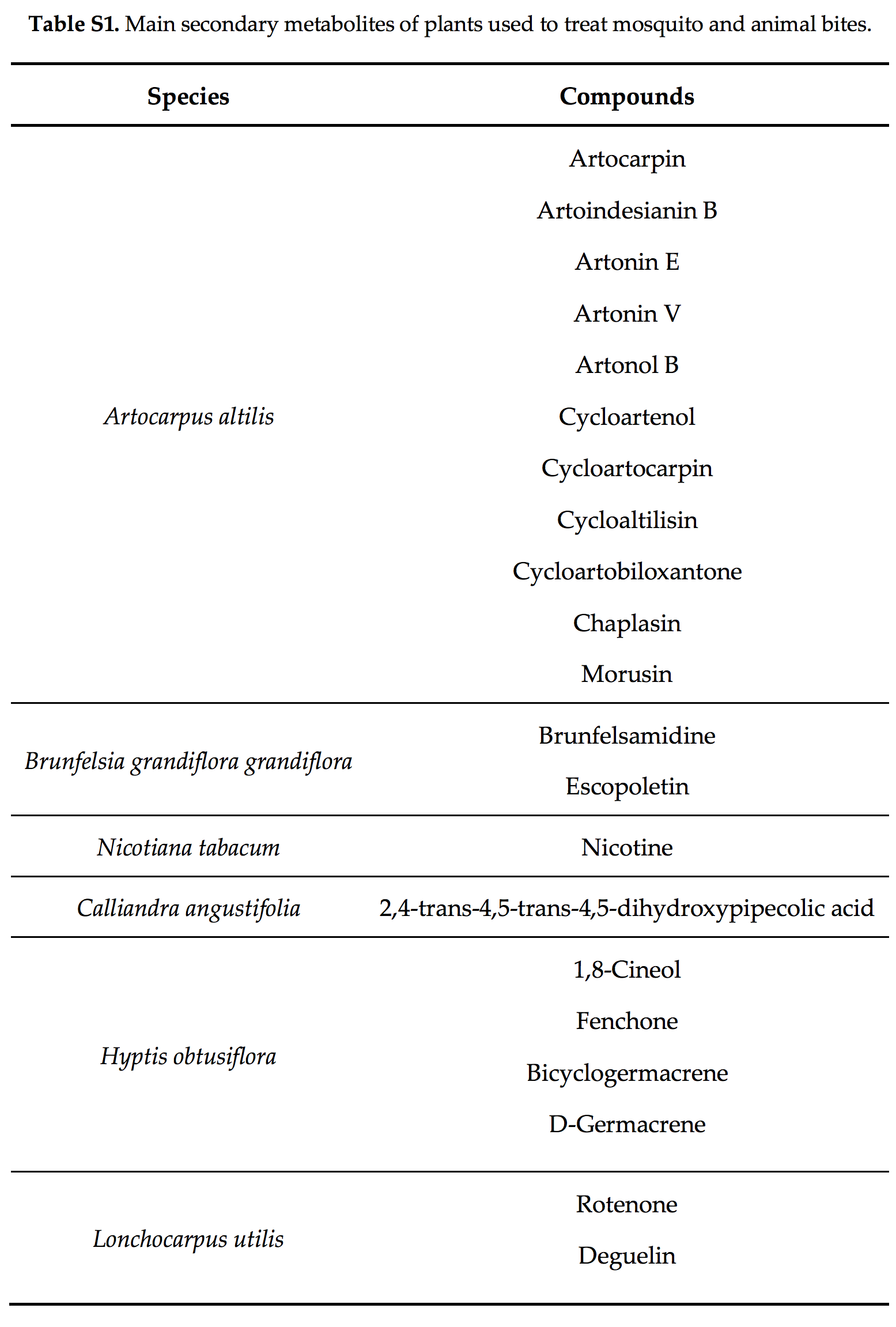

Supplement: Supplementary file 1 [file biology-11-00133-s001.zip › biology-1532271-supplementary/Table S1.tiff]
